# Reclassification of the taxonomic status of SEMIA3007 isolated in Mexico B-11A Mex as *Rhizobium leguminosarum* bv. *viceae* by bioinformatic tools

**DOI:** 10.1186/s12866-016-0882-5

**Published:** 2016-11-04

**Authors:** Luciano Takeshi Kishi, Camila Cesário Fernandes, Wellington Pine Omori, João Carlos Campanharo, Eliana Gertrudes de Macedo Lemos

**Affiliations:** Departamento de Tecnologia, Laboratório de Bioquímica de Microrganismos e Planta – LBMP, UNESP - Universidade Estadual Paulista, Faculdade de Ciências Agrárias e Veterinárias, Via de Acesso Prof. Paulo Donato Castellane s/n, 14884-900 Jaboticabal, SP Brazil

**Keywords:** Genome sequencing, Core genome, Comparative analysis, Ortholog genes, Phylogenetic analysis

## Abstract

**Background:**

Evidence based on genomic sequences is extremely important to confirm the phylogenetic relationships within the *Rhizobium* group. SEMIA3007 was analyzed within the *Mesorhizobium* groups to define the underlying causes of taxonomic identification. We previously used biochemical tests and phenotypic taxonomic methods to identify bacteria, which can lead to erroneous classification. An improved understanding of bacterial strains such as the *Mesorhizobium* genus would increase our knowledge of classification and evolution of these species.

**Results:**

In this study, we sequenced the complete genome of SEMIA3007 and compared it with five other *Mesorhizobium* and two *Rhizobium* genomes. The genomes of isolated SEMIA3007 showed several orthologs with *M. huakuii*, *M. erdmanii* and *M. loti*. We identified SEMIA3007 as a *Mesorhizobium* by comparing the 16S rRNA gene and the complete genome.

**Conclusion:**

Our ortholog, 16S rRNA gene and average nucleotide identity values (ANI) analysis all demonstrate SEMIA3007 is not *Rhizobium leguminosarum* bv. v*iceae*. The results of the phylogenetic analysis clearly show SEMIA3007 is part of the *Mesorhizobium* group and suggest a reclassification is warranted.

**Electronic supplementary material:**

The online version of this article (doi:10.1186/s12866-016-0882-5) contains supplementary material, which is available to authorized users.

## Background

Rhizobia is the collective name of the genera *Rhizobium*, *Sinorhizobium* and *Mesorhizobium*, which are soil and rhizosphere bacteria of agronomic importance because they form nitrogen-fixing symbioses with leguminous plants [1, 2]. Thus, rhizobia are considered bio-fertilizers and have been used as inoculants for over 120 years. Rhizobial genetic diversity and the plant-bacteria molecular interactions have been well-studied [3]. The growth rate of *Mesorhizobium* is intermediate between the genera *Rhizobium* and *Bradyrhizobium* and is one of the largest genera. Additionally, the *Mesorhizobium* genera consists of 24 species found in Asia, Europe, the Mediterranean region and Africa [4, 5].

Jarvins et al. [6] were the first to request the creation of the *Mesorhizobium* genus and reclassified several genera identified as *Rhizobium* into *Mesorhizobium*. The correct phylogenetic identification of a species requires an accurate technical characterization [7, 8].

The taxonomy of *Mesorhizobium* requires the reclassification of species because there is a need for studies to avoid classification problems. Taxonomic information provides access to basic trait information such as physiology, epidemiology and evolutionary history [9]. The correct taxonomic assignment of bacterial genomes is a primary and challenging task [10–13].

The partial 16S ribosomal RNA gene (16S rRNA) is a molecular marker widely used in the taxonomy of bacteria. However, this gene has no consensus sequence to correctly classify microorganisms at the species level [14–16]. Thus, DNA-DNA hybridization (DDH) has been used as the gold standard for defining prokaryotic species at the genomic level. DDH is the only taxonomic method that offers a numerical and relatively stable species. Therefore, DDH influences how the current classification system has been constructed [17].

DDH is an expensive and laborious method that is available in only a few laboratories worldwide, since it requires the hybridization of hundreds of strains and often does not resolve the taxonomic problems. However, it is an important limiting factor for the description of new species, particularly in countries with the greatest biodiversity. Prokaryotic species continue to be a group of strains due to DNA-DNA re-association values greater than 70 % [14, 18].

The recent development of sequencing technologies has enabled us to carefully assess microbial communities by generating many nucleotide sequences at lower costs. Next generation sequencing (NGS) technologies have revolutionized the field of microbial ecology and allows researchers to determine the level of diversity more closely using in-depth sequencing. There are various applications using these NGS platforms, which range from single-gene targeted sequencing to whole-genome sequencing and shotgun metagenome sequencing [19]. With the availability of whole genome sequences, the gene content based approaches appear promising in inferring the bacterial taxonomy. The complete genome sequencing of a bacterial genome often reveals a substantial number of unique genes present only in that genome which can be used for its taxonomic classification [11, 12].

The recent improved access to various new gene sequences and the definition of prokaryote species has led to doubts regarding the suitability of the DNA-DNA hybridization method [20]. The new proposals include the analysis of several genes or the entire genome. One proposed analysis method is to analyze common genes between two strains and determine the average nucleotide identity values (ANI). An ANI value exceeding 94 % corresponds to 70 % traditional DNA-DNA hybridization [21, 22].

This analysis method also considers genes with ecological functions. Other ANI values suggested replacing 70 % hybridization with 95 % ANI and 69 % conserved DNA. In the protein coding portion of the genome, these values would suggest 85 % conserved genes [23]. The most recent proposals recommend > 95–96 % ANI to delineate species and would replace the traditional 70 % cut off threshold used for DDH sequences [17].

The aim of this study was to evaluate SEMIA3007 isolated in Mexico as B-11A Mex and is classified by phenotypic taxonomic methods such as *Rhizobium leguminosarum* bv. *viceae* by different groups of researchers. We used a combination of complete 16S rRNA sequencing and complete genome analysis to reclassify B-11A as *Mesorhizobium* sp.

## Results and discussion

### Bacterial growth curve

**Fig. 1:**
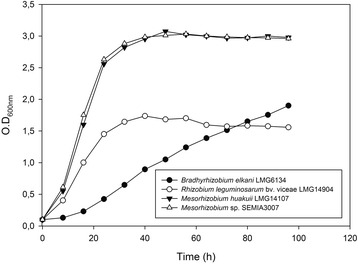
Bacterial growth curve. *Bradyrhizobium elkanii* LMG6134, *Rhizobium leguminosarum* bv. v*iceae* LMG14904, *Mesorhizobium huakuii* LMG14107 and *Mesorhizobium* sp. SEMIA3007 strains

The bacterial growth curve of SEMIA3007 is shown in Fig. 1. SEMIA3007 grew similar to the median strains of *Rhizobium*, *Mesorhizobium* and *Bradyrhizobium*. These findings phenotypically characterize SEMIA3007 as part of the genus *Mesorhizobium*. This strain was originally isolated in Mexico (B-11A Mex) and classified taxonomically as *Rhizobium leguminosarum* bv. *viceae* SEMIA3007 by a combination of phenotypic methods, biochemical tests and partial sequencing of the 16S rRNA gene.

### Genome assembly of SEMIA3007 and its features

The sequencing result shows that strain SEMIA3007 has the following characteristics: one contig of 6,990,002 bp, G + C content 63 %, 6,814 coding sequences (CDS) and a total of 55 RNAs. In the SEMIA3007 genome, there are two clusters encoding nitrite reductase (nirV and nirK) and four clusters related to denitrification processes that reduce nitrate to nitrogen gas. It is postulated that after host infection this cluster is responsible for allowing *Brucella suis* to survive low oxygen concentrations because the cells can use nitrogen oxides as final electron acceptors [24, 25]. The presence of this pathway enables SEMIA3007 to use this mechanism of intracellular survival during host infection.

We also found the following other genes were present in the genome of SEMIA3007: *nif*A, *nif*S, *nif*U, IscA-like, *nif*B, *frd*N, *nif*X, *nif*X2, *nif*E, *nif*N, *nif*Q, *nif*W, *nif*H, *nif*D, *nif*K, *nif*Z and *nif*T. These results suggest mechanisms for denitrification processes. The *nif* genes function in the transformation of ammonia nitrogen, nitrate, and nitrite ammonification and code for proteins such as nitrate reductase (EC 1.7.99.4), nitrite reductase [NAD(P)H] (EC 1.7.1.4), ferredoxin-nitrite reductase (EC 1.7.7.1) and nitrite reductase (cytochrome; ammonia-forming) (EC 1.7.2.2).

The SEMIA3007 genome also contains a subsystem for assimilation of ammonia and the bacteria can use ammonia assimilated for metabolism of amino acids (glutamate). The system uses glutamate ammonia ligase (EC 6.3.1.2), glutamate synthase (NADPH) (EC 1.4.1.13), glutamate synthase (NADH) (EC 1.4.1.14) and glutamate synthase (ferredoxin) (EC 1.4.7.1).

System secretions of type I, type II, type IV and type VI were identified. These system components are common among rhizobia. The type IV secretion systems are identified in microorganisms associated with plants and are usually composed of Vir proteins [26]. The operon of the type IV SEMIA3007 system features 12 genes encoding the following proteins: VirB1-VirB4, VirB6, VirB8-VirB9, VirB11, VirD4 and VirG. The virB region is responsible for coding key virulence factors in the symbiosis species *Mesorhizobium* [26, 27]. This operon may assist in inducing acidification of the phagosome in the cells after phagocytosis. The acidification may lead to the segregation of unknown effector molecules and create changes in the host cell endosome that generate a new intracellular compartment in which the attacker can replicate [28]. The secretion systems of types III, IV and VI and the nodulation factors are considered responsible for lead host specificity in *Mesorhizobium huakuii* [4].

### Genome comparisons of SEMIA3007 and *Rhizobium*

**Fig. 2:**
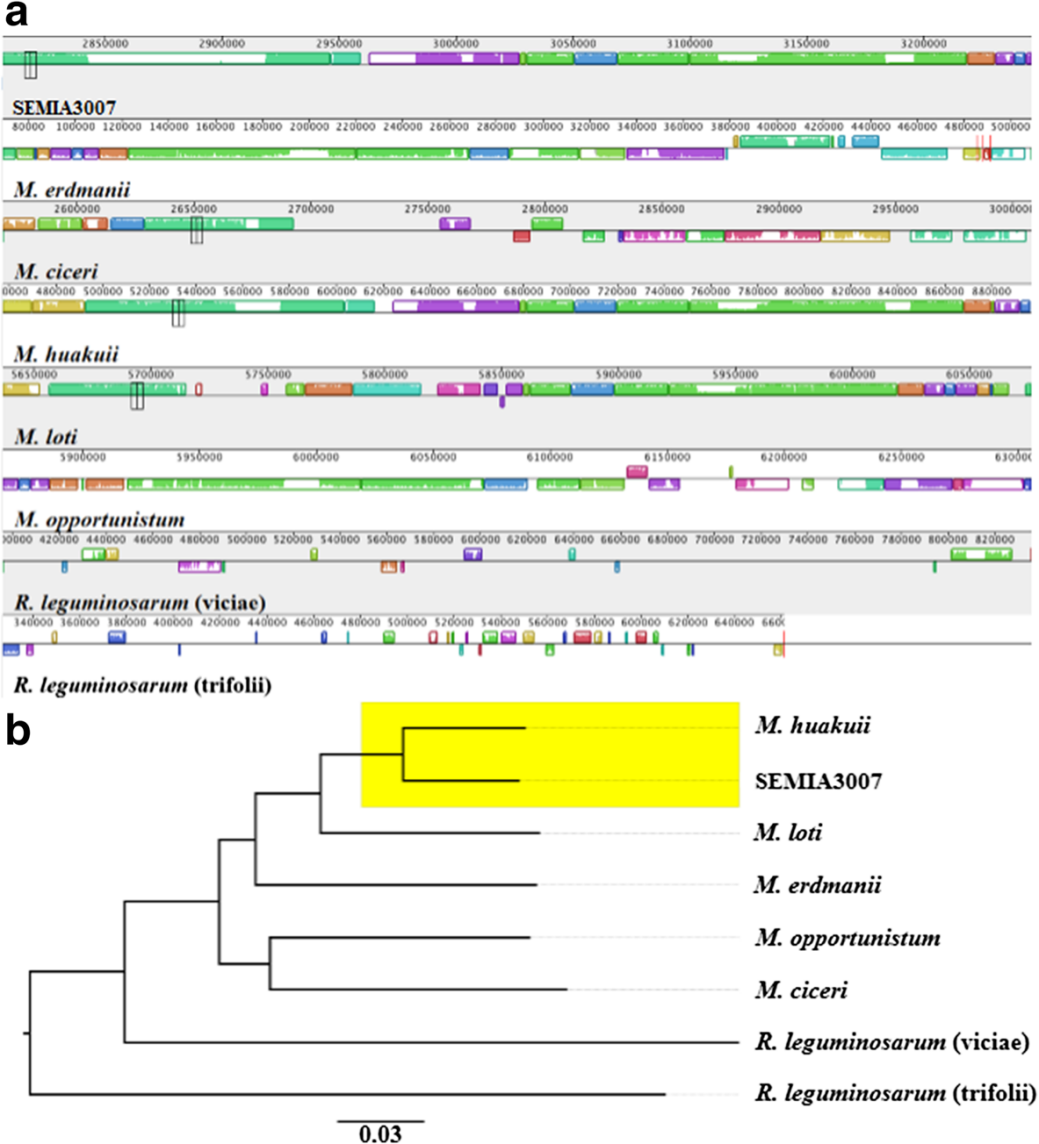
Genomes comparison between *Mesorhizobium* and *Rhizobium*. **a** A genome alignment of eight genomes using Mauve reveals collinear blocks conserved (LCB) among all genomes. Each chromosome is shown horizontally and homologous blocks in each genome are shown as identically colored regions. **b** Similarity between genomes aligned by Mauve program showing the phylogenetic relationships between genomes

Our analysis of the similarity between genomes can be used to differentiate microorganisms. We used the genomes of SEMIA3007, *Rhizobium leguminosarum* bv*. viceae* (gi 115254414), *Rhizobium leguminosarum* bv. *trifolii* (gi 240861949), *Mesorhizobium erdmanii* (gi 548692182), *Mesorhizobium ciceri* (gi 317165637), *Mesorhizobium huakuii* (gi 657121522), *Mesorhizobium loti* (gi 47118328) and *Mesorhizobium opportunistum* (gi 336024847) to construct a progressive alignment using the program Mauve. We found there was a high degree of similarity (block synteny and direction) between SEMIA3007 and the *Mesorhizobium* group and a limited number of blocks collinear between *Rhizobium* (Fig. 2).

ANI [23] is one method that has replaced the DDH [29], and it is the best *in silico* parameter representing DDH that has been experimentally demonstrated [22, 29]. Our genome comparisons for taxonomic purposes were based on BLAST calculations [30]. An ANI value of 95 % ± 0.5 % identity corresponds to 70 % DDH [23], which is a value often recommended to delimit species when used in conjunction with other criteria, such as phenotypic traits [31].

Richter and Rossello-Mora [17] describe a software tool (JSpecies) designed to easily allow the calculation of ANI based on the BLAST algorithm [30] and the MUMmer ultra-rapid aligning tool [32]. We also calculated the tetranucleotide frequencies, which are alignment-free parameters that have been successfully applied to phylogenetically sort metagenome inserts [33]. Therefore, the 95–96 % ANI threshold can be readily used as an objective boundary for species circumscription if it is reinforced by high TETRA correlation values [17]. Our results demonstrate that SEMIA3007 is more genetically similar to *Mesorhizobium huakuii* than *Rhizobium* (Table 1).

**Table 1:** Probability of pairwise comparison

	Genome (bp)	%GC	Gene	%ANIb	%ANIm	%Tetra
SEMIA3007	6990002	62.9	7173	-	-	-
*M. huakuii*	6364365	63.2	5838	98.45	98.94	99.97
*M. loti*	7036071	62.7	7043	93.46	94.64	99.96
*M. opportunistum*	6884444	62.9	6418	87.31	89.17	99.85
*M. ciceri*	6264489	62.5	6100	85.70	87.25	99.85
*M. erdmanii*	7018265	62.7	6491	88.89	90.40	99.86
*R. leg_viciae*	5057142	61.1	4797	70.72	83.54	95.75
*R. leguminosarum*	7418122	60.7	7293	70.27	82.79	96.01

Nucleotide identity values (ANI) and correlation indexes of their Tetra-nucleotide signatures between SEMIA3007 with *Mesorhizobium* and *Rhizobium*

### Phylogenetic analysis using 16S rRNA

The results of sequencing the 16S rRNA gene SEMIA3007 were subjected to a membership analysis taxonomy in RDPII bank. We utilized the classifier tool with a threshold of 95 %. The result showed the identity was 100 % *Mesorhizobium*. Additionally, there was 100 % identity with the 16S Ribosomal RNA database using the Blast program (June 2006).

A phylogenetic analysis was performed using data available on the NCBI database to assess whether SEMIA3007 should be identified and cataloged as *Rhizobium leguminosarum* bv. *viceae* within *Rhizobium* or be reclassified as part of the *Mesorhizobium* group (Additional file 1: Table S1).

**Fig. 3:**
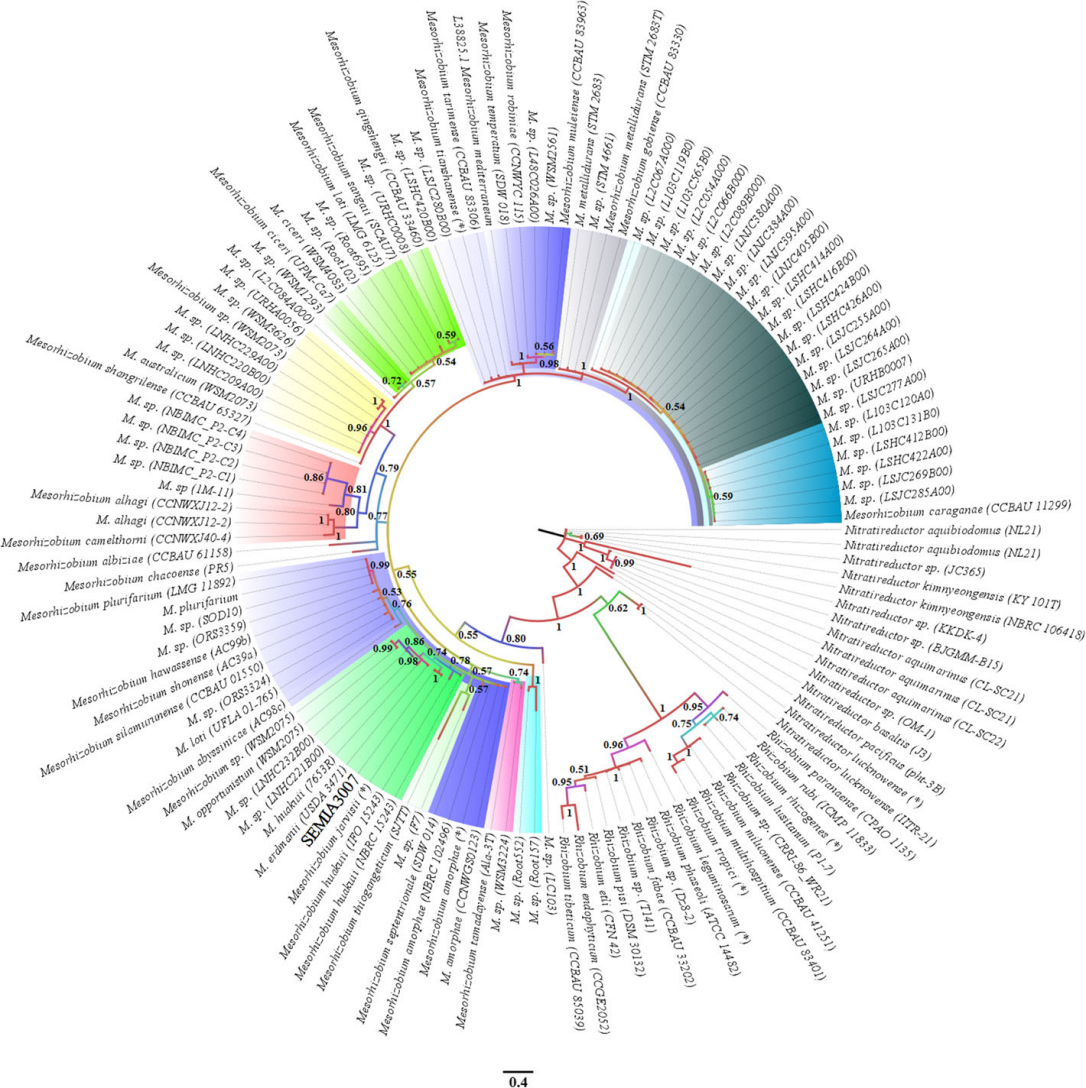
Phylogenetic tree showing the taxonomic position of SEMIA3007 strain between groups of *Mesorhizobium* and *Rhizobium*. Genetic differences between bacteria of 0.4 %. The numbers in the branches show the probability calculated by MrBayes with the colors

The results of the phylogenetic analysis clearly show SEMIA3007 is a member of *Mesorhizobium* and is separate from the *Rhizobium* group, which suggests a reclassification of SEMIA3007 is warranted (Fig. 3).

### Comparison of gene orthologs

**Fig. 4:**
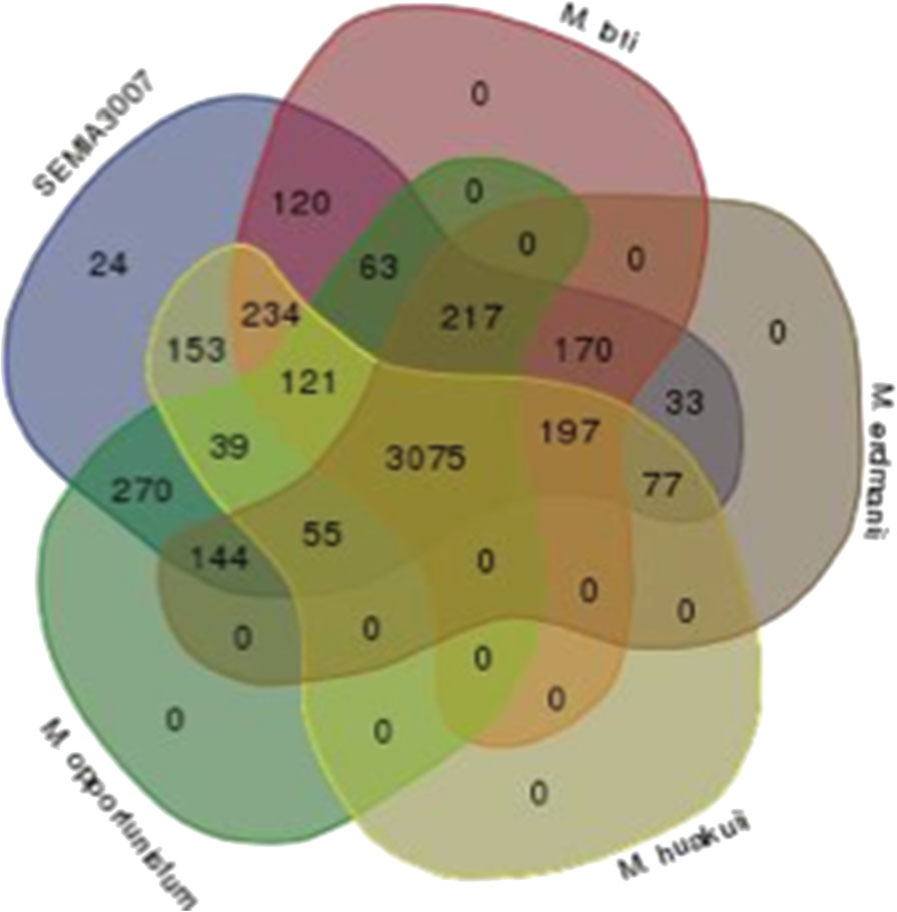
Venn diagram showing core genome analyses of *Mesorhizobium* strains. The number of protein-coding gene ortholog sharing among five *Mesorhizobium*. SEMIA3007; *M. huakuii* (CP006581.1*)*; *M. loti* (NC_002678.2); *M. opportunistum* (NC_015675.1); *M. erdmanii* (NZ_AXAE01000048.1)

Previous studies have compared genes to differentiate organisms. We used OrthoMCL clustering to identify “core genes”, which are the number of unique and shared orthologs of SEMIA3007 and *Mesorhizobium* (Fig. 4). A total of 32,604 proteins from SEMIA3007 (6,814 proteins), *M. huakuii* (5,838 proteins), *M. erdmanii* (6,491 proteins), *M. loti* (7,043 proteins) and *M. opportunistum* (6,418 proteins) were evaluated. We used an inflation index of 1.5 to complete genes and identified 3,075 ortholog groups within the five genomes.

The clusters of orthologs in Fig. 4 show there are 3,075 ortholog groups in SEMIA3007 representing 69.5 % of the total CDS in the genome. However, SEMIA3007 and *M. huakuii* showed 3,951 (79.1 %) common ortholog groups. We found that SEMIA3007 and *M. erdmanii* shared 4,392 (87.9 %) orthologs. There were 4,197 (84 %) orthologs in common between SEMIA3007 and *M. loti*. There were also 3,984 (79 %) orthologs shared between SEMIA3007 and *M. opportunistum*. Therefore, isolated SEMIA3007 shows a large number of *Mesorhizobium* gene orthologs. These findings suggest that SEMIA3007 is a *Mesorhizobium* strain*.*

Therefore, the results for growth curve of SEMIA3007, comparative analysis of the genome, ANI, gene orthologs and phylogenetic analysis using 16S rRNA show that SEMIA3007 is not *Rhizobium leguminosarum* bv. *viceae* suggesting its reclassification for Mesorhizobium group [10–13].

## Conclusions

NGS technologies have proven their utility in genomic and metagenomics areas since their earliest application appeared in 2006. Identifying each individual sequence is important in microbial community analysis because the taxonomic information provides access to basic trait information such as physiology, epidemiology and evolutionary history. The taxonomic information also permits indirect inference of their ecological roles in a given environment [19].

Whole-genome sequencing has proven to be valuable and critical for refining the phylogenetic positions and correct taxonomic classification of rhizobial strains [10, 11, 34]. In this study, we sequenced, assembled and annotated the SEMIA3007 genome. We used this genome sequence to examine the phylogenetic relationship between *Mesorhizobium* and *Rhizobium* genus. SEMIA3007 was classified by phenotypic taxonomic methods and biochemical tests as *Rhizobium leguminosarum* bv. *viceae*. However, our results strongly suggest that SEMIA3007 belongs to the *Mesorhizobium* genus. The placement of SEMIA3007 in a *Mesorhizobium* genus is supported by our analysis of ANI, ortholog genes and phylogenetic analysis.

We can see a high degree of similarity and block synteny and direction between SEMIA3007 and the *Mesorhizobium* group. Our results demonstrated there were a limited number of blocks collinear between *Rhizobium*. Additionally, the ANI based on a pairwise genome comparison of all shared ortholog protein coding genes is 98 % with *Mesorhizobium huakuii*. Our phylogenetic analysis demonstrated that SEMIA3007 is not part of the *Rhizobium* genus, and the ortholog genes revealed sufficient ability to identify SEMIA3007 as *Mesorhizobium*.

The concepts of orthology originated from the field of molecular systematics [35] and have recently been applied to functional characterizations and classifications on the scale of whole-genome comparisons [36–38]. In comparative genomics, the clustering of orthologous genes provides a framework for integrating information from multiple genomes by highlighting the divergence and conservation of gene families and biological processes.

The identification of orthologous groups in prokaryotic genomes has permitted cross-referencing of genes from multiple species and has facilitated genome annotation, protein family classification, studies on bacterial evolution and the identification of strains. The ultimate goal of taxonomy is to construct a classification that is operative and predictive for any discipline in microbiology. The classification is also essentially stable for old and new strain such as Rhizobia and the collective names of the genera *Rhizobium*, *Sinorhizobium*, *Mesorhizobium*.

## Methods

### Bacterial growth curve

The strains of *Bradyrhizobium elkanii* LMG6134, *Rhizobium leguminosarum* bv. v*iceae* LMG14904, *Mesorhizobium huakuii* LMG14107 and *Mesorhizobium* sp. SEMIA3007 were cultured for 96 h with shaking (150 rpm) at 30 °C in TY medium [39] in triplicate. To obtain the bacterial growth curve, the OD reading was collected every 8 h.

### Bacterial strain and DNA preparation

SEMIA3007 was cultured for 48 h at 28 °C with 145 rpm shaking in TY medium [39]. The SEMIA3007 cells were harvested by centrifugation, and the total DNA was prepared using a Wizard® Genomic DNA Purification Kit (Promega).

### Sequencing and annotation of the genome

The *de novo* sequencing of the SEMIA3007 genome used a combined strategy involving Illumina – HiscanSQ. The libraries were constructed using a TruSeq® DNA Sample Prep kit and Nextera Mate Pair Sample Preparation kit (Illumina®). The cluster formation of library templates was performed with the TruSeq PE Cluster kit v3 (Illumina®) and the Illumina cBot workstation using conditions recommended by the manufacturer. Paired end 100 base pair (2x100bp) sequencing by synthesis was performed with TruSeq SBS kit v3 (Illumina®) on an Illumina HiscanSQ using protocols defined by the manufacturer. The base call conversion to sequence reads was performed using CASAVA 1.8.3 (Illumina®). As a result, paired-end and mate pair fastq files were trimmed using Scythe 0.991 (https://github.com/vsbuffalo/scythe), Cutadapt 1.7.1 [40] and the quality of data was filtered by Prinseq program [41] with Phred ≥20. The sequence assembly was performed using the Spades 3.6.1 program [42]. The prediction of ORFs and annotation were performed using the Rast system [43].

### Genome comparisons and average nucleotide identity (ANI)

For comparing the genome of SEMIA3007 to others genomes we compute an alignment of the six genomes, we used the Progressive Mauve algorithm [44]. An alignment of the four *Mesorhizobium* and *Rhizobium* genomes was constructed using the default mauveAligner parameters. The resulting LCBs were inspected using the Mauve alignment viewer, and the minimum LCB weight was adjusted to eliminate LCBs consisting of only repetitive elements (LCB Weight 600).

Reference genomes for comparison purposes were retrieved from the GenBank database (http://www.ncbi.nlm.nih.gov/genbank/). Sequences were uploaded into the JSpecies software package (http://www.imedea.uib.es/jspecies) to perform pairwise genome calculations of the average nucleotide identity (ANI) [17, 23] and support the proposed cut-off level of 95 % as a species delineation threshold [22].

### Ortholog analysis

The ortholog groups in multiple genomes can be useful for annotation and revealing the patterns of phylogenetic proteins from different strains. The groups also provide insights into the evolutionary conservation and diverse cellular functions in different species.

Four coding sequences (CDS) from genomes/drafts of *Mesorhizobium loti*, *Mesorhizobium huakuii*, *Mesorhizobium erdmanii* and *Mesorhizobium sp.* were extracted from GenBank files (Additional file 1: Table S1), representing four species (five with SEMIA3007 CDS). The pan and core genome analysis was conducted by determining shared (homologous) and species-specific protein-coding genes using OrthoMCL [36] with e-value cutoff 1 × 10^−20^, protein percent identity ≥50 % and MCL inflation of 1.5. OrthoMCL computes families of homologous genes for pan and core genome analyses. The families in which two or more genomes participate were used to determine numbers plotted. OrthoMCL was run with blast e-value cut-off of 1e-5 and an inflation parameter of 1.5. The table with orthologs was used to plot Venn diagrams (http://bioinformatics.psb.ugent.be/webtools/Venn/) [36].

### 16S rRNA gene sequencing

The amplification of the 16S rRNA gene of the SEMIA3007 was performed with FD1 and RD1 primers [45]. The PCR reaction mixture consisted of 30 ng of DNA, 7.5 pmol of each primer, 0.2 mM of dNTPs, 1.5 mM of MgCl_2_, Buffer 1X and 2.5 U Taq DNA polymerase (Ludwig Biotec). A thermocycler model PTC-100 ™ Programmable Thermal Controller (MJ Research, Inc.) was used with a thermal profile of 96 °C for 2 min, 40 cycles of 96 °C for 30 s, 53 °C for 1 min and 60 °C for 4 min. After the PCR reaction, the products were purified with a Wizard® SV Gel and PCR Clean-Up System (Promega). The amplicon was sequenced with 1 μl of BigDye Terminator v3.1, buffer 0.75X (Tris-HCl 200 mM, pH 9.0 and MgCl2 5 mM), 10 pmoles of primer FD1, 50 ng of DNA and sterile Milli-Q distilled water (10 μL q.s.p). Sequencing was performed on Sequencer ABI PRISM 3130xl DNA Analyzer (Applied Biosystems) following the manufacturer’s instructions.

### Downloading the sequences 16S rRNA in GenBank

The National Center for Biotechnology Information (NCBI) was used to search the genome for species *Mesorhizobium* (March 15, 2016). All complete gene sequences for *16S rRNA* (16S ribosomal RNA) were downloaded from GenBank (Additional file 1: Table S1) [46].

### Phylogenetic analysis of 16S rRNA gene

The 16S rRNA gene set were aligned using the MAFFT v7.215 program [47]. The search for the best nucleotide substitution matrix was performed with the Phangorn package [48] in R [49] and the feature modelTest. The construction of a phylogenetic tree was performed with the Mrbayes v3.2.2 program [50] using the matrix replacement General Time Reversible (GTR) with gamma variation (G) and invariable sites (I) with 10.000.000 generations. The best evolutionary model was chosen based on Akaike information criterion with correction (AICc).

### Nucleotide sequence accession number

The data sets results of this article are available in the NCBI BioProject SRR3703040.

## Additional file

Additional file 1: Table S1. Complete genomes and drafts. (XLSX 18 kb)

## Abbreviations

16S rRNA: 16S ribosomal RNA gene
AICc: Akaike information criterion with correction
ANI: Average nucleotide identity values
CDS: Coding sequences
DDH: DNA-DNA hybridization
DNA: Deoxyribonucleic acid
GTR: General time reversible
LCB: Collinear blocks conserved
LCB: Locally collinear blocks
NAD(P)H: Nitrite reductase
NADH: Glutamate synthase
NADPH: Glutamate synthase
NCBI: National Center for Biotechnology Information
NGS: Next generation sequencing
OD: Optical density
ORF: Open read frame
PCR: Polymerase chain reaction
TY: Triptone, yeast medium
